# Effect of mineral filler on low velocity impact behaviour of bi-directional glass fibre/epoxy composites exposed to varied temperature conditions

**DOI:** 10.1371/journal.pone.0322208

**Published:** 2025-06-09

**Authors:** K.S Lokesh, C G Ramachandra, Shrinivasa Mayya.D, T Varuna, Praveen Kumar Kanti, Abhinav Kumar, Melkamu Biyana Regasa

**Affiliations:** 1 Srinivas Institute of Technology, Mangalore, Karnataka, India; 2 Presidency University, Bangalore, Karnataka, India; 3 University Center for Research & Development (UCRD), Chandigarh University, Mohali, Punjab, India; 4 Department of Nuclear and Renewable Energy, Ural Federal University Named after the First President of Russia Boris Yeltsin, Ekaterinburg, Russia; 5 Department of Mechanical Engineering and Renewable Energy, Technical Engineering College, The Islamic University, Najaf, Iraq; 6 Centre for Research Impact & Outcome, Chitkara University Institute of Engineering and Technology, Chitkara University, Rajpura, Punjab, India; 7 Chemistry Department, College of Natural and Computational Sciences, Wollega University, Nekemte, Ethiopia; National Chung Cheng University, Taiwan & Australian Center for Sustainable Development Research and Innovation (ACSDRI), AUSTRALIA

## Abstract

Fibre reinforced plastics are predominantly used in automotive, aerospace applications due to their promising features of low cost, easy of processing and durability, however these composites are more susceptible to varied temperature conditions under different loading conditions. One such problem where these composites vary greatly in their performance is due to impact loading conditions. The objective of present research is to determine the effect of calcium inosilicate (also termed as wollastonite) powder which is used as a filler on low velocity impact (LVI) behaviour of bi-directional glass fibre reinforced with epoxy thermosets (GFRP) for different loading percentage of 1%,3%,5% and 7%. Composites were prepared through manual lay-up route and prepared samples were exposed for 3 different temperature conditions of continuous heating and cooling cycles at +50^0^c, −5^0^C including room temperature conditions for 168hrs. Aged samples were tested for low velocity impact(LVI) testing by keeping constant energy of 30J. Results revealed that addition up to 3% filler influence largely on LVI response of GFRP composites for all the temperature conditions, addition to this polynomial regression of 9^th^ order was performed and the results were best fit with experimental values. Fractography study revealed the severity of damage modes upon LVI testing.

## 1. Introduction

Traditional composites were advanced with wide spectrum of its applications. Refining the reinforcements and matrix through filler occupy the huge space to delve in to materials of unique features. The trend of advancing composite grabs the global attention from utilizing waste resources as fillers. Starts from red brick dust [1], Kota stone dust [2], clamshell waste [3], Tamarind seed powder, eelgrass, and adamant creeper [4], Prosopis juliflora bark powder [5], LD sludge [6], witness the potential and role of fillers in enhancing the mechanical performance of polymer composites. Though natural fibres and their hybridizing effect remains as exciting categories from both mechanical and tribological aspects [7–9]. Applications pertaining to Aerospace industry is keen to utilize most selective candidates like glass fibre thermosets as predominant key structural elements due to their exceptional durability and strength especially with operating temperature of range from −54^0^c to +55^0^c [10]. Range may even low as −80^0^c as well as go high while at higher altitudes [11]. Various Damage mode mechanisms to report cracks in matrix, delamination with fibre splitting are severe failure modes for developed structures [12–16]. Barrier to impact damage is majorly influenced by temperature due to which its effects are quintessential to understand damage behaviour. Fibre matrix failure both can occur in same time when impact behaviour comes in to picture [17,18]. When impacting done on aeronautical structures, properties quoting brittle-ductile behaviour need to be explored for high and lower temperatures [19]. However, studies reported with more cross-linked polymers exhibits restricted impact loading [20–23]. Two of most damages due to impact and bending are noted because of thin lamina undergo delamination and for compact laminates ends with broken fibres fibre [24]. Recent study reports composites made of bi-directional glass fibre laminates witness excellent impact resistance rather uni-directional laminates [25,26]. Temperature can further influence on sustainability of GFRP composites during impact through toughness and brittleness [19]. Mechanical properties along with energy absorption capacity which directs failure mechanisms are due to change in glass transition temperature which in turn due to High and low temperatures [27]. Polymers operating slightly high temperature nearing to their Glass transition range which is considered to be most crucial. Though operating temperature meant for the comfortability range of thermosets, but for aeronautical applications its quite higher [28] which leads to reduction in stiffness nearing Tg. Temperature varied between −60 °C to 20 °C alters the impact performance of GFRP plates even tested at low energy level of 20J [29], importantly, thermally aged, LVI tested, CFRP composites at constant energy level witness larger damage area rather non -aged samples [30]. But still few studies enumerate, reduction in damage size with deflection as the range of temperature decreases [31]and also delamination zone is considerably reduced when GFRP composites were aged hygrothermally [32]. Significant observation been made in tightening of reinforcements by enhancing fibre matrix bonding through contractions in epoxy matrix for temperature reaching cryogenic range [31], its proven that thermal aging significantly affects the LVI behaviour of GFRP composites report from the study [33]. Also, drastic changes in perforation are found due to LVI of GFRP plates for lower temperatures [29]. Though slight increase in Tg for calcium inosilicate filler loaded with epoxy matrix the GFRP composites [34], with best of author’s knowledge its necessary to evaluate the damage mechanism incurred through low velocity impact testing for the said composite plates developed through epoxy modified with calcium inosilicate filler when subjected to different temperature conditions of -5c, + 50c and compare the damage effect with room temperature conditions for fixed energy level.

## 2. Materials and methodology

### 2.1. Materials

**Fig 1:**
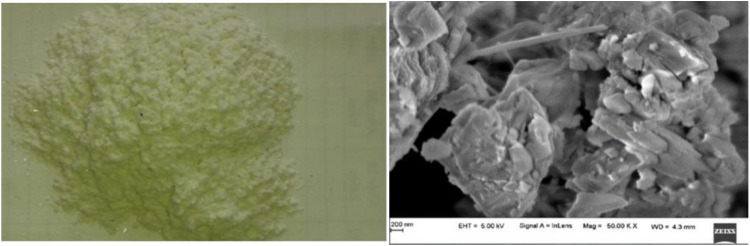
(a) Calcium inosilicate mineral filler used in the present study & (b) Microstructure of filler used.

**Fig 2:**
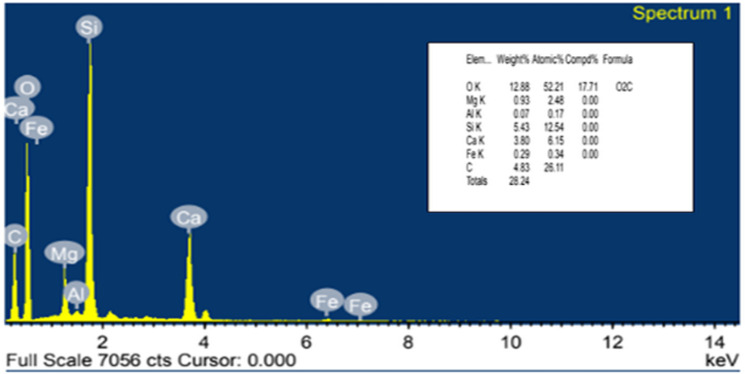
Elemental composition of filler used in the present study.

Bi-directional glass fibers with aerial density of 360gsm which is used as reinforcement whereas Epoxy resin of grade LY556 with the density of 1.2g/cc was used as matrix in the present study. Both fibre and matrix materials were supplied from S&S polymers, Bangalore, Mineral filler required for the present study of commercial grade was procured from Mahaveer mineral supplier from Rajasthan. Micro structure of filler as depicted in Fig 1 and elemental composition with calcium and silicon as major constituents as shown in Fig 2. As received calcium ino silicate filler properties are discussed in below Table 1.

**Table 1:** Properties of calcium inosilicate powder.

Colour	White
**pH**	9.9
**Hardness:**	4-5 Mohs
**Density**	2.94 g/cc
**Specific gravity**	2.87-3.09
**Gran size**	250µ
**Molecular formula**	CaSiO_3_
**Chemical Composition**	48.28% CaO & 51.72% SiO_2_

### 2.2. Methodology

#### 2.2.1. Sample preparation.

**Fig 3:**
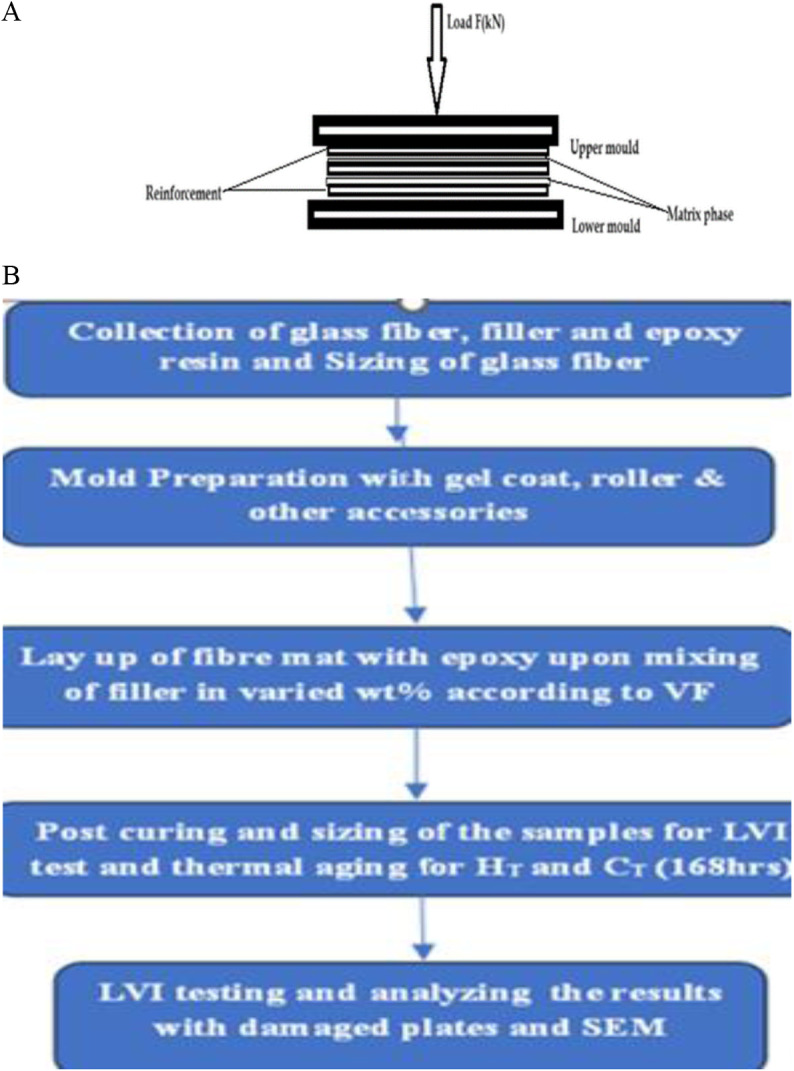
(a) Compressing the stacked layers. (b) Flow chart of sample preparation method.

Composites laminates were developed with the size of 250x250mm. Volume fraction of 60:40 ration is employed for preparation of the samples with different filler loading percentage of 1,3,5,7% by weight. Epoxy and hardener ratio was 10:1 was maintained for sample preparation. Before hardening, calcium inosilicate is weighted to specified weight percentage of 1,3,5,7% with respect to matrix followed by mixing of filler with epoxy has been carried out manually. Using stirrer, powdered filler is infiltrated with epoxy by manual stirring from slower to moderate speed to ensure uniform mixing of filler. Composite plates were then prepared though hand layup route followed by compressing with maximum load of 5kN on the stacked laminates as shown in Fig 3a and the process flow for sample preparation is represented from the Fig 3b. UV-curing of the prepared composite samples were done for 48hrs to ensure effective curing of the samples followed by sizing of the samples.

#### 2.2.2. Drop weight impact test.

Drop weight impact testing method is widely used among other testing methods to determine the low velocity impact on polymer composites subjected to the various conditions of drop weight test. The test has been conducted for low acceleration to understand real word sudden loading conditions. Drop weight impact test samples are specifically developed as per the requirements of testing standards prescribed for the device, requirements usually in terms of sample mounting to final impact damage. Obtained results for the samples tested for room temperature aged are compared with other two sets of samples which are exposed to two different temperature conditions, one with heating cycle of +50^0^c where the samples are kept in hot air oven chamber and other with cooling cycle of −5^0^c where the samples are kept in freezing chamber fixing the ageing time for both set for 168hrs. Maximum heating cycle temperature was considered upon reported glass transition temperature of the present composite samples [34]and Ageing temperature duration of thermal ageing has been followed according to the study [33].

The low velocity drop weight impact testing device comprising data acquisition unit, load cell equipped with semiconductor strain gauge and velocity measurement devices equipped with optical sensors with transmitter and receiver. The steel dart of radius 10 mm with hemispherical head is used for the impacting the samples. Specially equipped fixtures to clamp the samples are designed to ensure clamping force via preloaded hemispherical springs and load cell is installed to record the variational responses of load with time, displacement to be recorded. GFRP composites prepared with no filler and with varied composition of calcium -inosilicate filler via manual layup route and test samples are made suitable to carry out the drop weight impact test as per the prescribed standards. These samples are clamped on the fixture bed with slot of 100 × 100 mm. Drop weight impact test was carried out with specified velocity of 3.5m/s with fixed mass of 6.2 kg and falling height of 0.5m Technical specifications of the device are tabulated in Table 2.

**Table 2:** Technical Specifications of LVI device.

Maximum drop height	5.8m
Maximum velocity	10.77m/s
Maximum capacity	6960J
Maximum specimen dimension	200*200 mm

**Fig 4:**
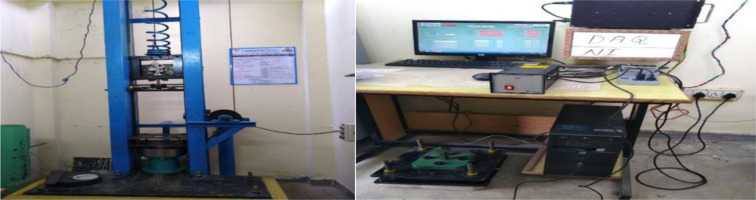
(a) Drop weight Impact testing device (b) Output measuring setup.

The low velocity impact characteristics has been discussed in detail in the results section. The current study aims to find the right composition of wollastonite filler and its effect on epoxy matrix system in combination with reinforcement and thereby takes up the maximum impact load for both normal and varied temperature conditions. The tests are carried out with a device low velocity falling weight testing machine which is as shown in Fig 4a and the output measuring device is as shown in Fig 4b. The impact energy can be regulated by adjusting the drop height or with the adjustment made in fixing the mass in kg of steel dart. For the present study the maximum height of 0.5m is chosen with fixed mass of 6.2 kg with expected impact energy of 30J. If we increase the drop height or mass the potential energy will also increase proportionally and the equal amount of energy will be converted in to kinetic energy.

## 3. Results and discussion

### 3.1. Drop weight impact test

For the present study samples are tested as per ASTMD 3029 [35]. Prepared samples were mounted on fixed slot with 100 mm × 100 mm which gives an area considered for assessing the LVI strength. The load taken by the samples prepared with different filler composition with respect to aged conditions are discussed in the below section. Prepared composite samples are clamped on the fixture bed with slot of 100 × 100 mm. The steel dart of radius 10 mm with hemispherical head is used for the impacting the samples. Drop weight impact test was carried out with specified velocity of 3.5m/s with fixed mass of 6.2 kg and falling height of 0.5m. Drop weight impact testing has been done for the varied filler composition of GFRP composite sample for 3 different sets of temperature conditions. The variations of load and acceleration over time for room temperature aged samples are presented in the below sections.

#### 3.1.1. Room temperature aged samples.

Samples with room temperature aged condition were subjected to LVI testing with the help of drop weight tower, impact characteristics such as Force, displacement, energy, acceleration, contact time are obtained and mean values are tabulated in the Table 3

**Table 3:** LVI test characteristics with their Mean Values for samples aged with R_T._

Filler Composition (%)	Force (kN)	Acceleration (m/s^2^)	Max Displacement (mm)	Max Energy (J)	Contact Time (mili-seconds)
0	4.8	840.08	12.5	11.31	8.9
1	4.9	851.89	10.1	13.11	6.9
3	5.8	872.5	14.7	16.29	11.9
5	4.9	839.7	12.4	14.49	8.7
7	3.9	800.36	12.1	10.89	6.9

**Fig 5:**
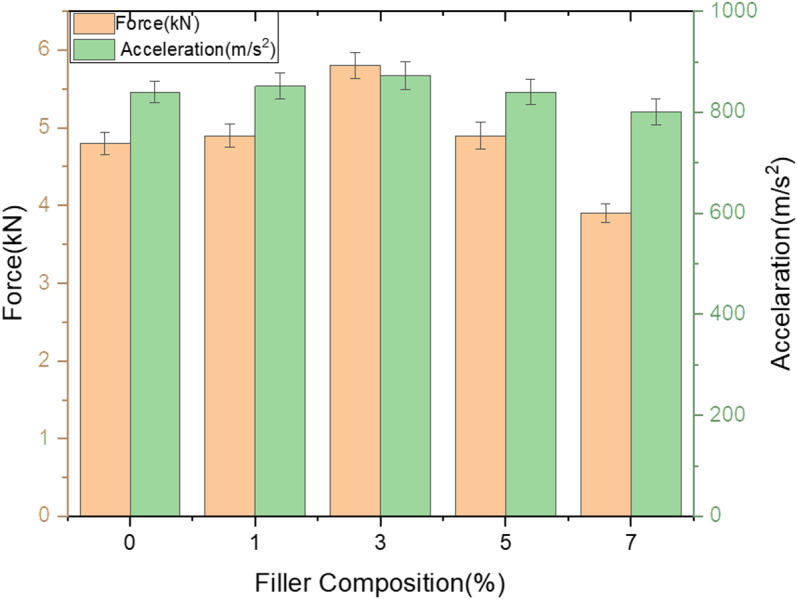
Variation of Load and acceleration over different filler composition.

***Effect of fillers on load bearing ability of GFRP composites*:** Below Fig 5 depicts the variation of load and acceleration over different filler composition. Effect of filler on load bearing capability was discussed for room temperature aged samples. Though the response against LVI for Neat composite in the present study is better as compare to previous study [36] filler percentage would influence the LVI characteristics of GFRP samples to greater extent. Increase in weight percentage from 4–6% of SMA filler to GFRP samples has increased impact resistance as compare to neat samples when exposed to LVI test with the same testing conditions [37].

It is declared result that 3% filler shows excellent resistance to low velocity impact damage and noted that 18% increase in load bearing capability compare to neat GFRP composite and upon loading further up to maximum percentage, 33% decrement in its resistance to LVI loading as compare to 3% composition and it’s clear that optimal loading percentage of wollastonite in epoxy matrix stands at 3% filler loading.

**Fig 6:**
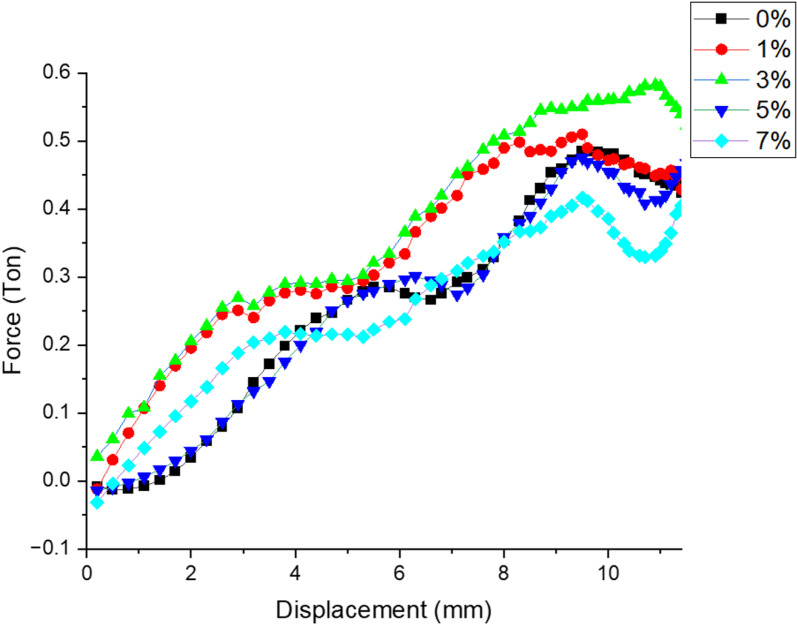
Variation of Load over Displacement for different filler composition.

***Effect of filler on load v/s displacement***: Fig 6 represents the variation of Load over Displacement for different filler composition when applied under room temperature. There is gradual increase in the load value is observed for the samples filled with 3% filler as compare with other combinations representing the saturation range of wollastonite to make matrix more resistance to low velocity impact loading. Considerable decrement in the said value is observed for 7% filler sample.

**Force and Contact Time for Room Temperature aged samples.**

**Fig 7:**
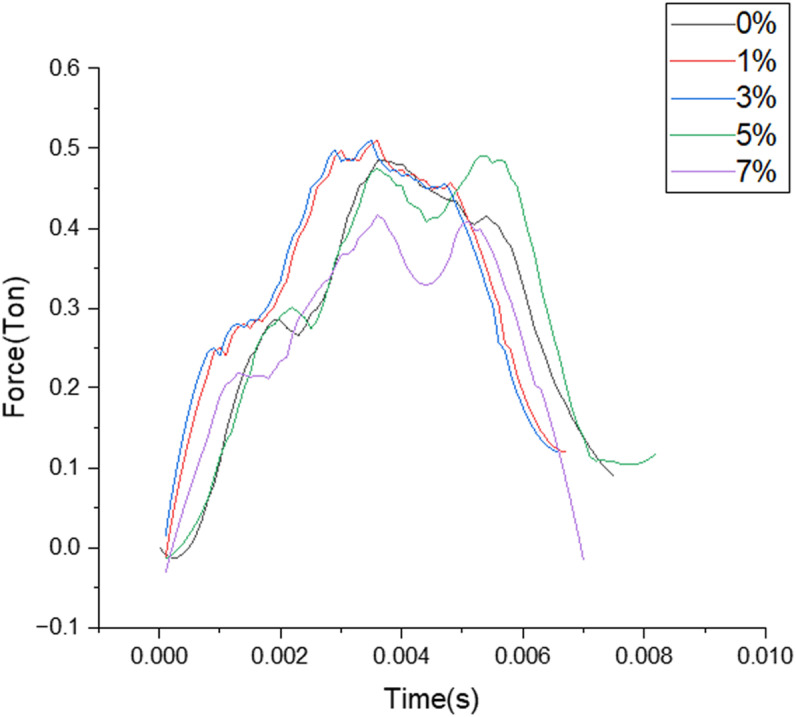
Plot of Load over contact time for different filler composition.

Contact time measured for different filler composition where the duration of contact measured in seconds are clearly depicted from the above Fig 7 for the different load bearing values reported when the samples aged at room temperature are subjected to LVI testing.

#### 3.1.2. Test results for heating cycle (+50^0^c).

Samples aged for continuous heating cycle of +50^0^c condition were exposed to LVI testing with the help of drop weight tower, impact characteristics such as Force, displacement, energy, acceleration, contact time are obtained and mean values are tabulated in the Table 4.

**Table 4:** LVI test characteristics with their Mean Values for samples aged at heating cycle(H).

Filler Composition (%)	Force (kN)	Acceleration (m/s^2^)	Max Displacement (mm)	Max Energy (J)	Contact Time (ms)
0	3.9	661.12	8.6	9	7.9
1	4.9	1101.98	11.1	12.42	7.4
3	6.8	1065.05	12.2	18.9	7.9
5	5.8	993.16	10.8	14.49	7.4
7	4.9	715.17	12.6	11.98	8.9

**Fig 8:**
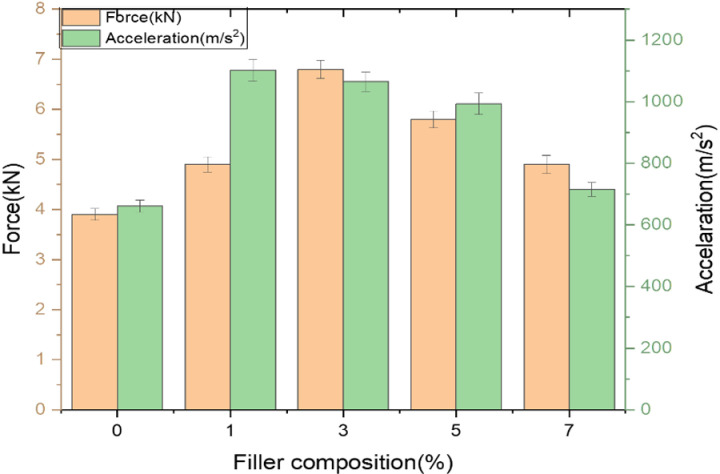
Variation of Load and acceleration over different filler composition.

***Effect of fillers on load bearing ability of GFRP composites***: Below Fig 8 depicts the variation of load and acceleration over different filler composition. Effect of filler on load bearing capability was discussed for samples subjected to heating cycle. It is repeatedly noticed that samples loaded with 3% filler shows excellent resistance to LVI damage and noted that 50% increase in load bearing capability compare to neat GFRP composite and upon loading further up to maximum percentage, 33% decrement in its resistance to LVI loading as compare to 3% composition and it’s clear that optimal loading percentage of wollastonite in epoxy matrix stands at 3% filler loading. Relevant study also reports that thermal aging significantly affects the LVI behaviour of GFRP composites [33].

**Effect of filler on Load v/s Displacement.**

**Fig 9:**
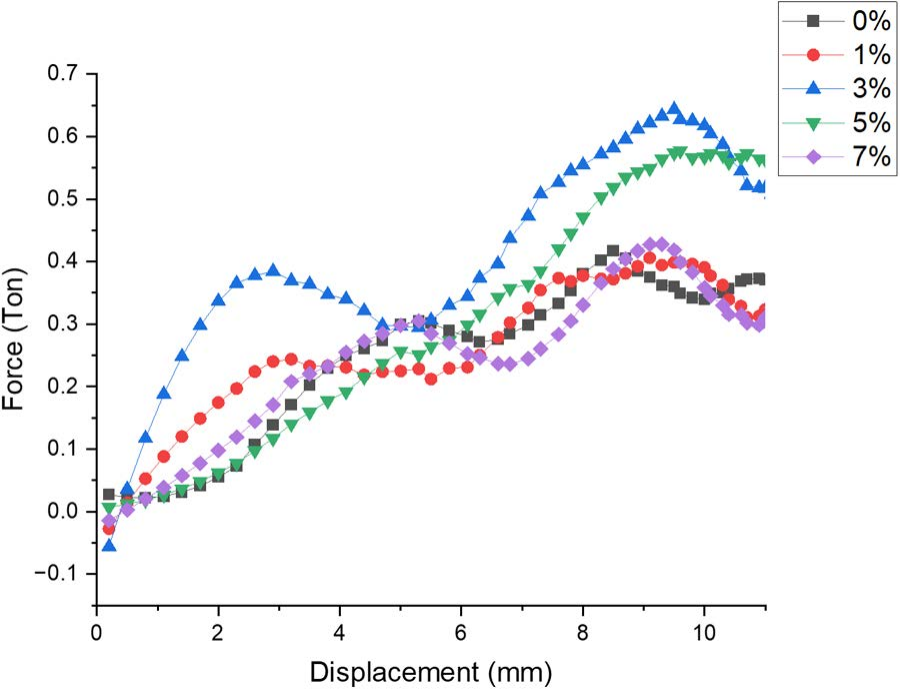
Variation of Load over Displacement for different filler composition.

Fig 9 depicts the effect of filler on load taking capability of material and corresponding displacement for all the compositions when applied under heating cycle of +50^0^C temperature. There is gradual increase in the load value is observed for the samples filled with 3% filler as compare with other combinations representing the saturation range of wollastonite to make matrix more resistance to low velocity impact loading. Considerable decrement in the said value is observed for 7% filler sample.

**Fig 10:**
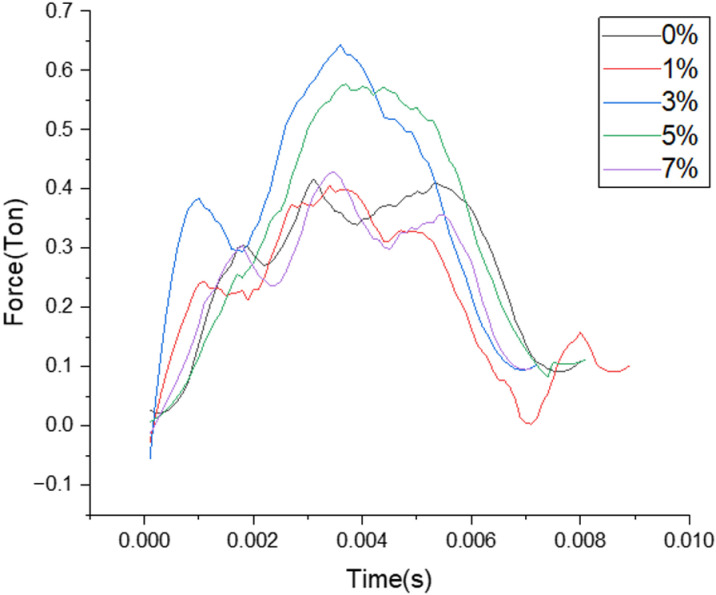
Plot of Load over contact time for different filler composition.

***Force and contact time for samples aged with heating cycle***: Contact time measured for different filler composition where the duration of contact measured in seconds are clearly depicted from the Fig 10 for the different load bearing values reported when the samples aged through continuous heating cycle are subjected to LVI testing.

#### 3.1.3. Test results for cooling cycle (−5^0^c).

Samples aged for continuous cooling cycle of −5^0^c condition were exposed to LVI testing with the help of drop weight tower, impact characteristics such as Force, displacement, energy, acceleration, contact time are obtained and mean values are tabulated in the Table 5.

**Table 5:** LVI test characteristics with their Mean Values for samples aged at cooling cycle(C).

Filler Composition (%)	Force (kN)	Acceleration (m/s^2^)	Max Displacement (mm)	Max Energy (J)	Contact Time (ms)
0	3.9	754.97	11.7	9.6	8.19
1	4.9	869.09	10.2	13.11	7.9
3	5.8	1027.2	14.6	17.64	8.9
5	4.9	928.89	10.8	13.68	7.8
7	3.9	745.1	10.1	10.02	7.2

**Fig 11:**
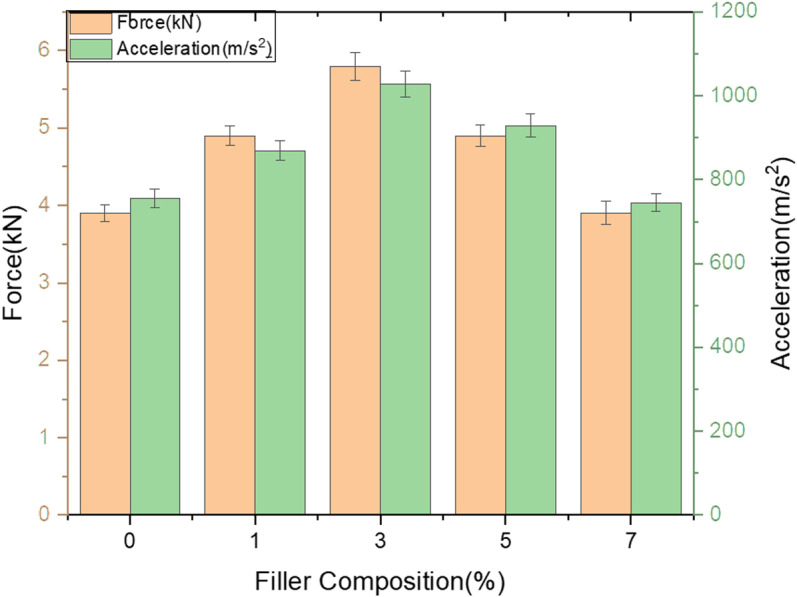
Variation of Load and acceleration over different filler composition.

***Effect of fillers on load bearing ability of GFRP composites***: Below Fig 11 depicts the variation of load and acceleration over different filler composition.

Effect of filler on load bearing capability was discussed for samples subjected to heating cycle. It is repeatedly noticed that samples loaded with 3% filler shows excellent resistance to LVI damage and noted that 33% increase in load bearing capability compare to neat GFRP composite and upon loading further up to maximum percentage, 33% decrement in its resistance to LVI loading as compare to 3% composition and it’s clear that optimal loading percentage of wollastonite in epoxy matrix stands at 3% filler loading. It is also observed that the trend of drop down in LVI is similar to that of other two set of thermal aging cycles.

**Fig 12:**
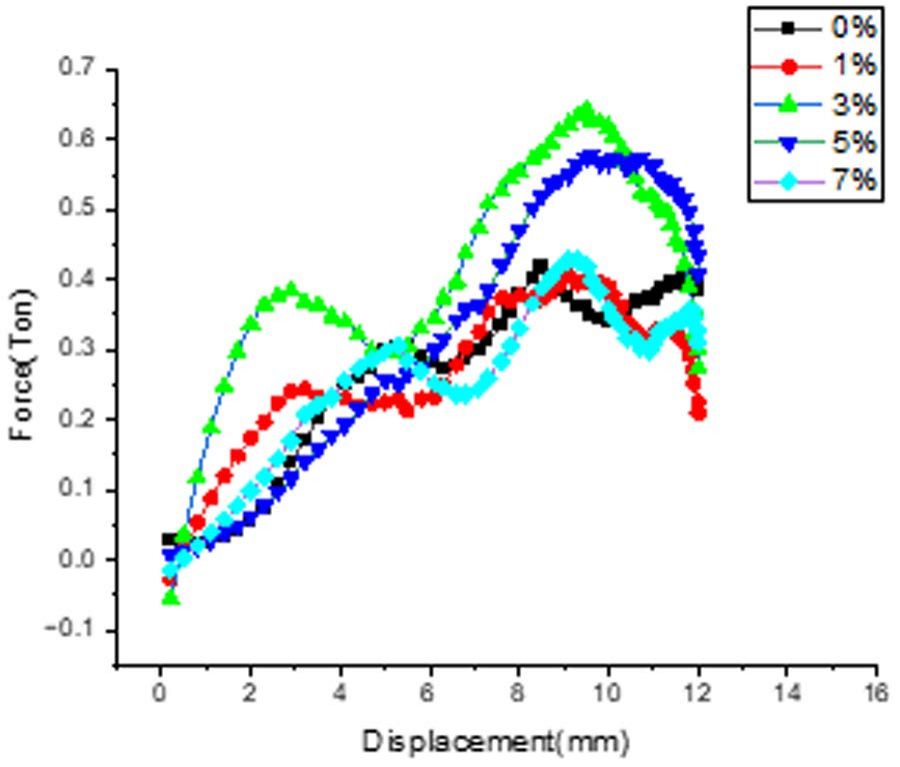
Variation of Load over Displacement for different filler composition.

***Effect of filler on load v/s displacement***: Fig 12 depicts the effect of filler on load taking capability of material and corresponding displacement for all the compositions when applied under continuous cooling cycle temperature of −5^0^c. There is gradual increase in the load value is observed for the samples filled with 3% filler as compare with other combinations representing the optimal loading range of wollastonite to make matrix more resistance to low velocity impact loading. Considerable decrement in the said value is observed for 1% filler sample.

**Force and Contact Time for samples aged with Heating cycle.**

**Fig 13:**
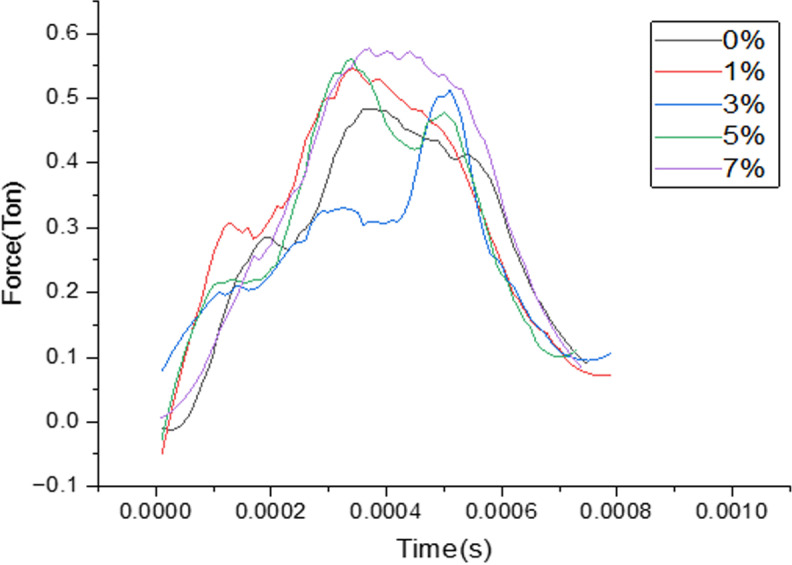
Plot of Load over contact time for different filler composition.

Contact time measured for different filler composition where the duration of contact measured in seconds are clearly depicted from the Fig 13 for the different load bearing values reported when the samples aged through continuous cooling cycle are subjected to LVI testing.

### 3.2. Damage analysis

**Fig 14:**
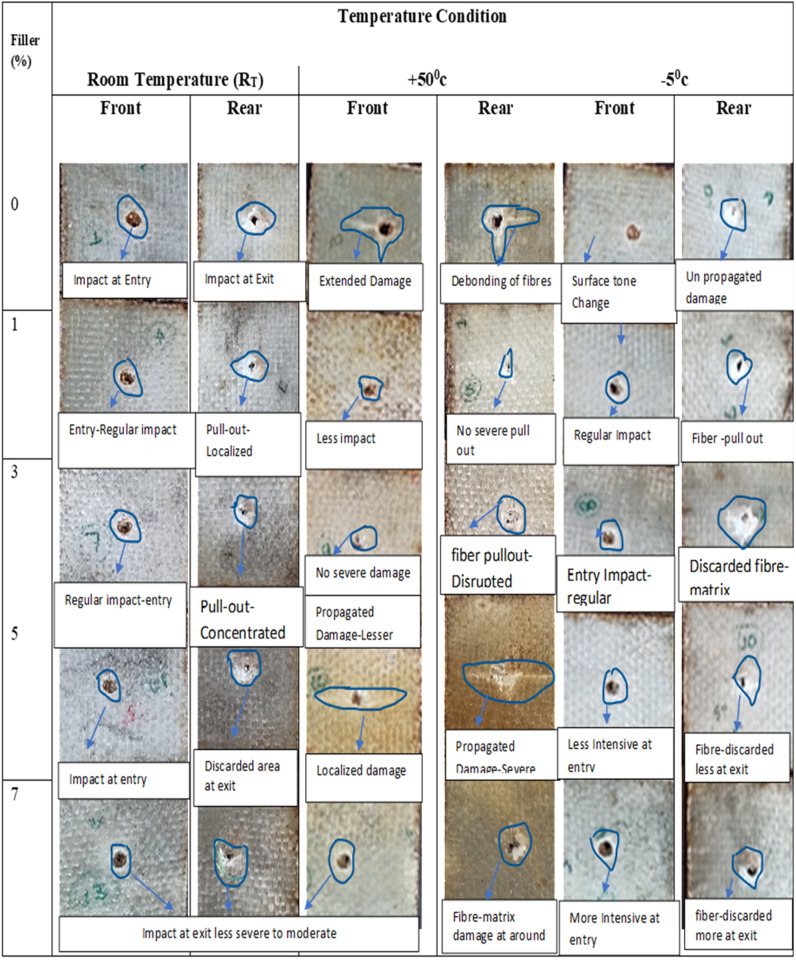
Damage modes for different filler and temperature conditions.

**Fig 15:**
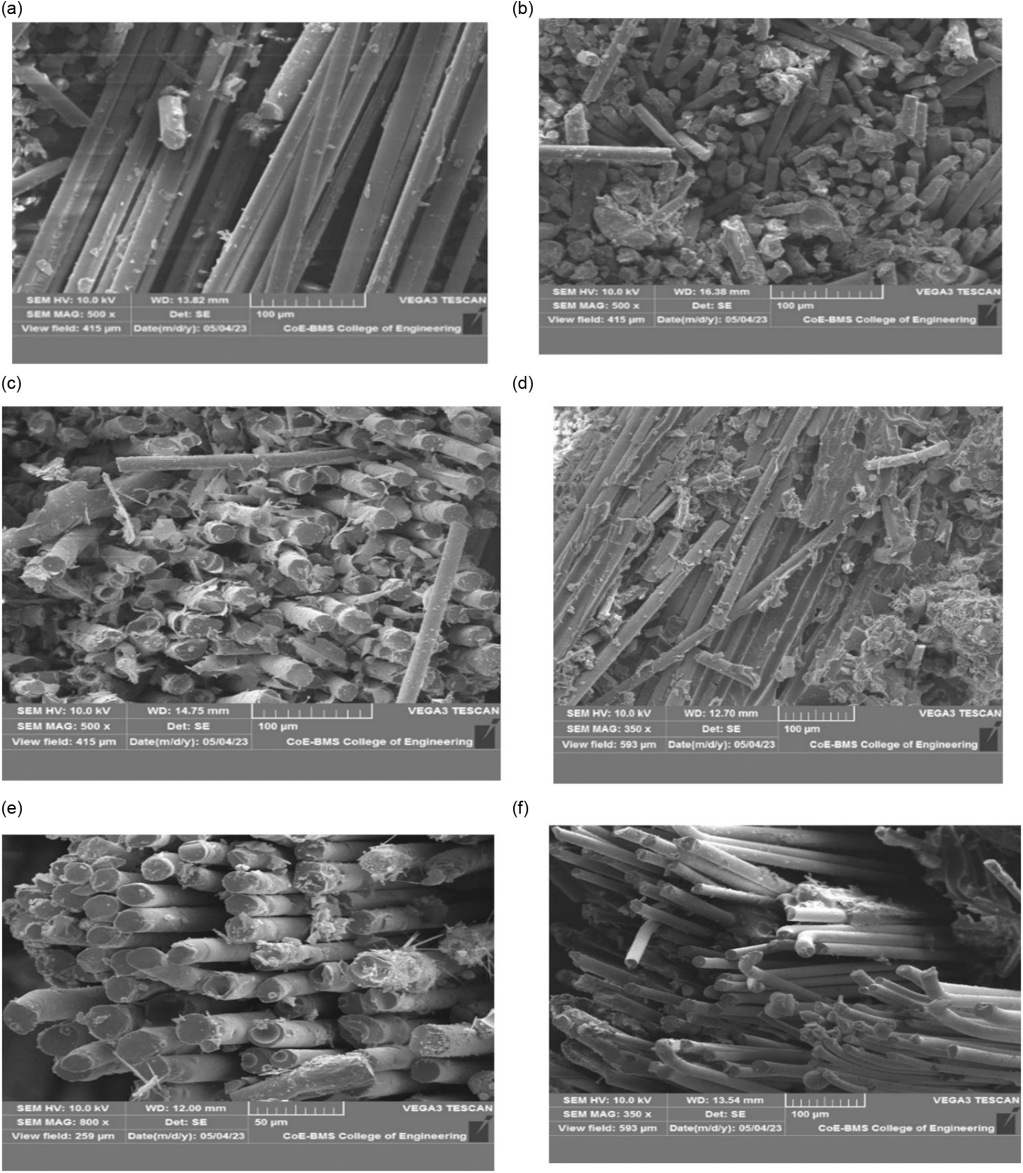
Fractured surface of (a) 3% filler at RT, (b) 7% filler at RT, (c) 3% at HC and (d) Neat Sample at HC (e) 3% at CC and (f) Neat Sample at CC.

Damage analysis of different filler composition for the room temperature condition has been studied. Analysis has been done both with the help of visible damage mode and microstructure images. Fig 14 depicts the visible damage zones for both front and rear sides. Formed dents against LVI for each sample with entry and exit was represented. Addition to sample with no severe damage to propagated damage was also figured out to understand the amount of energy absorbed with applied force and contact time was examined. For heating cycle its noted that visible changes in surface tone for aged samples been observed and matrix fibre pluffing as well as brittle damage mode for cooling cycle due to chilling effect is also noticed. Fig 15a and b depicts the micro structure of samples loaded with 3% and 7% filler. The damage mode observed for the post low velocity impact testing of the laminates is breakage of fibres and causing surface damage due to LVI loading. Sample loaded with 3% filler depicts better load taking capability due to the fact the addition of said range of filler maintains better compaction through better binding with matrix and makes the surface tougher than the other with lower load taking value for the sample with 7% filler leads to severe surface damage hence broken fibres. Addition of more filler content results in agglomeration of the matrix and ends with weaker matrix. LVI failure modes of different filler composition for the continuous heating cycle of +50^0^c has been studied. Fig 15c and d represents the micro structure of samples loaded with 3% and neat sample. The damage mode observed for the post low velocity impact testing of the laminates is fibre pull out and matrix crack observed.

From the experimental results it is observed that samples loaded with 3% filler experience comparatively lower damage to the surface against LVI loading due to the fact that fibres are adequate exposure of reinforcing fibres over the region of load applied resulted upon fibre fracture through fibre pull out was observed indicating the limiting value of filler of 3%, enhance the fibre-matrix binding characteristics as well continuous curing cycle improves the the better load bearing ability of the sample where the neat sample experience severe damage to the surface against LVI loading reporting lower load value results in matrix damage and fibre breakage is observed to more extent which may limit its ability to take up more load. Damage modes for different temperature conditions with varied filler compositions of the tested samples are detailed in the Fig 14.

LVI failure modes of different filler composition for the continuous cooling cycle of -50c has been studied. Fig 15e and f depicts the micro structure of samples loaded with 3% and neat sample. The damage mode observed for the post low velocity impact testing of the laminates is fibre pull out and bending edge of fibres. From the experimental results it is observed that samples loaded with 3% filler experience comparatively lower damage to the surface incurred against LVI loading due to the fact that fibres are exposed uniformly over the region of load applied resulted with complete fibre pull out was observed indicating optimal value of filler addition in to the matrix to enhance better bonding characteristics of matrix with reinforcement. Due to fact that the samples were exposed to continuous cooling cycle upon LVI loading samples with 3% filler fails completely in brittle mode due to chilled effect of matrix whereas neat GFRP laminates reports lower load value with the fibre pull out and bending edge like fibres indicating failure underwent through less brittle mode as compare to that of 3% filler. In fact, less damage severity has been proved for non-thermally aged CFRP composites as compare to aged samples [30] Its observed that neat GFRP sample fig(a) resembles bonding between fibre filaments are weak which are not adhered effectively as compare to optimal 3% where these particles act as entanglement points presenting the complex morphology when fibres are broken [36].

### 3.3. Influence of filler conditions for different temperature conditions

**Fig 16:**
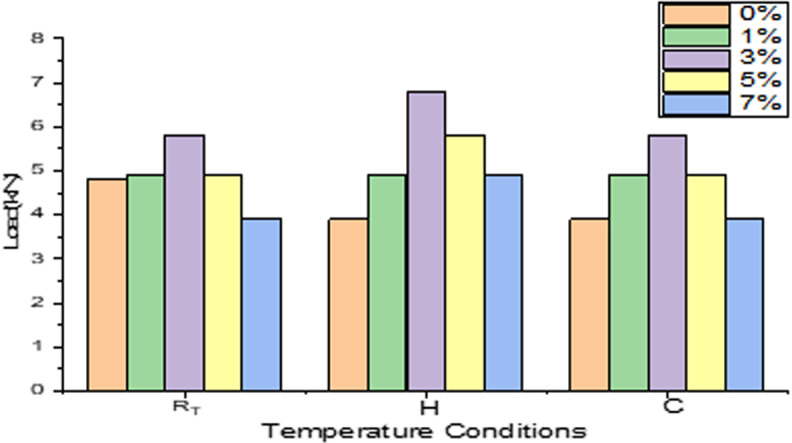
Influence of filler for 3 different temperatures.

Fig 16 represents the influence of wollastonite on load bearing capability of composites exposed to varied temperature conditions. For normal temperature condition, it is observed for 3% sample, shows excellent response to LVI damage and noted that 18% increase in its load bearing capability compare to neat GFRP composite and reports 33% decrement upon loading further to maximum range. And also due to the fact that if sample tested without filler once temperature is increased beyond R_T_, neat composite reports lower LVI strength as resin layers loses its strength while nearing its Tg [38,39].

Composites subjected to heating cycle of +50^0^c. It’s reported that samples loaded with 3% sample records excellent resistance to LVI damage and reports nearly 50% spike in its value compare to neat GFRP composite and there is 33% decrement in its value is observed when the samples are loaded with maximum range. For cooling cycles of −5^0^c, significantly 3% filler records better resistance to LVI damage value of 33% increase as compare to control sample and the same percentage dip is observed while reaching the maximum percentage of wollastonite. From the experimental results it’s clearly concluded that adding more filler to epoxy matrix beyond the optimal percentage leads to agglomeration and hence results in poor binding properties end up with reduced resistance to loading conditions. By consolidating for three different conditions, its concluded that effect of filler contributes greatly in enhancing the load bearing capability of the GFRP composite. For all different filler compositions, samples with 3% filler dominates for the all the temperature conditions.

### 3.4. Influence of temperature conditions on load bearing ability of different samples

**Fig 17:**
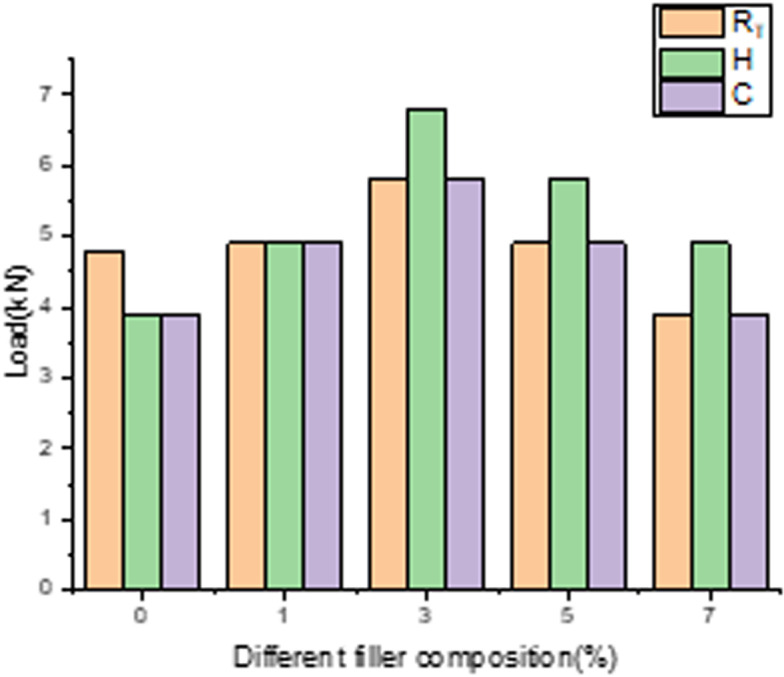
Influence of filler on different temperatures.

Fig 17 highlights the temperature effect on load taking capability of varied filler percentage. For room temperature aged conditions,samples loaded with 3% filler reports higher laod bearing ability as compare to rest of the combinations concluding the optimal loading conditions for room temperature conditions. For contineous heating & cooling cycle, the same trend has been observed for the samples loaded with 3% filler repeatedy reports its dominance againest LVI which is considered to be better accomodating percentage within the matrix to consistantly improve the load bearing capability of GFRP composites irrespective of temperature condtions.To consolidate for overal higher values of load, contineous heating cycle contributes softening of matrix due to contineous heating and then improve better bonding characteristics with reinforcement and its also reported from the preveous study that once samples exceeds +50^0^c till it reaches 80^0^c there is gradual decriment in its impact strenght with optimal volume fraction of fibre [40]. Its observed that for no filler composite that they reported better LVI characteristics at R_T_ than heating cycle due to the fact that once matrix nearing glass transition zone reports subsequent reduction in its LVI strength as resin layers reports reduced compressive strength which leads to comparitivly lower LVI strength [38,39].

### 3.5. Effect of filler and temperature on percentage of energy absorption

**Fig 18:**
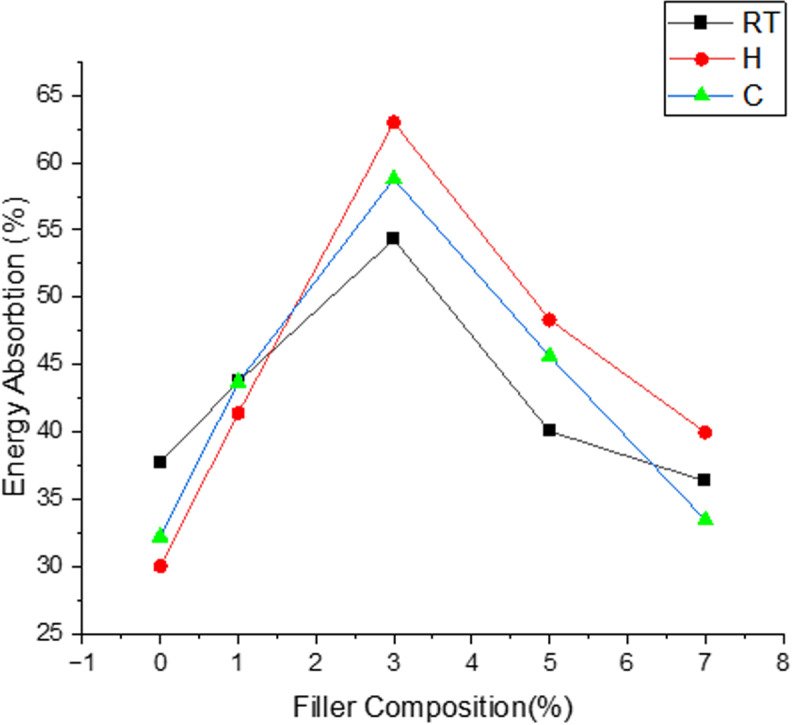
Effect of filler and Temperature on Percentage of Energy Absorption.

Below Fig 18 depicts the percentage of energy absorption for all the compositions subjected to different temperature ageing conditions which are analysed under drop weight impact testing condition by keeping the constant energy applied value of 30J where input parameters like load, drop height, size and shape of impact tester are remain unvaried. Experimental observations clearly shows that samples loaded with 3% filler performed with excellent energy absorption value for all the temperature conditions which reports the energy absorption range of 54–63% of which is about 31–54% higher value than that of neat composites for three temperatures. Considerable drop in energy absorption for R_T_ samples compare to thermally aged samples is due to the fact that continuous heating cycle contributes softening of matrix as enhanced curing due to heating cycle imparts binding characteristics with reinforcement and it’s also reported from the previous study that once samples exceeds + 50c till it reaches 80^0^c there is gradual decrement in its impact strength with optimal volume fraction of fibre [40].It is found from the results that effect of filler contributes more towards improving energy absorption capability of samples start from 1 to 3% but beyond 3% the energy absorption range follows downtrend for further loading of filler indicating that more filler content leads to agglomeration in the matrix which results in underperforming of samples for the energy absorption and load taking capability.

### 3.6. Regression analysis

**Fig 19:**
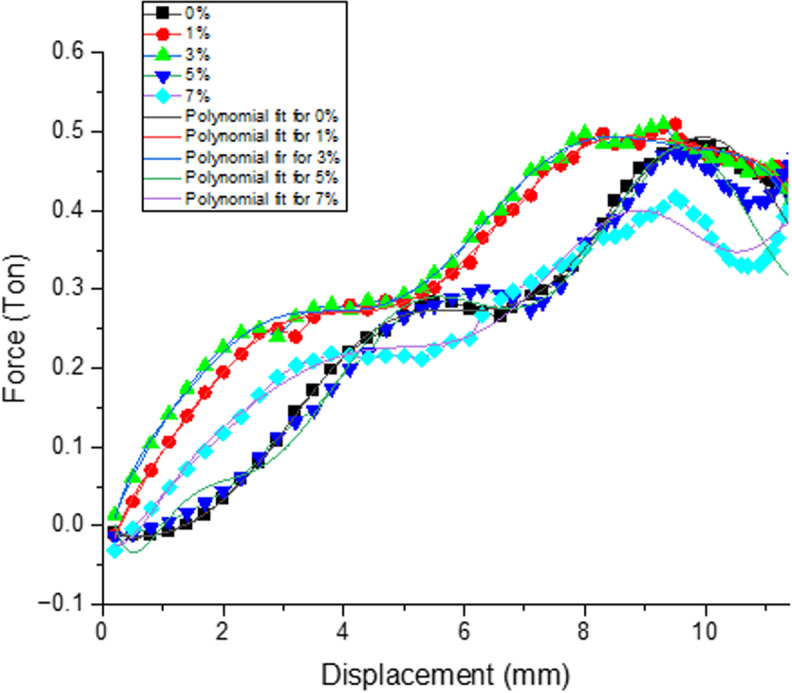
Correlation plots of experimental and regression values.

**Fig 20:**
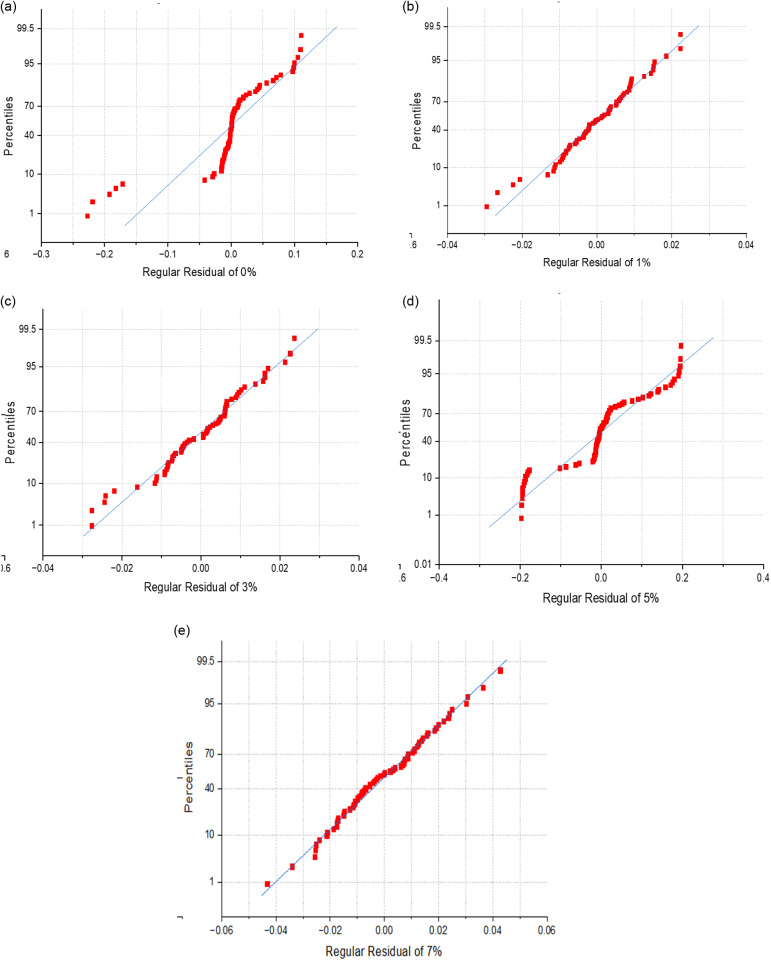
Regression characteristics of room temperature tested samples.

In order to validate experimental results of LVI test, regression modelling analysis of up to 9^th^ order was performed. Out of 3 temperature conditions, experimental values obtained for room temperature conditions were considered for the study. In order to check the nature of fit, normal probability plots, all the tested samples under the said condition were considered for regression study. Polynomial regression with lower order remains insignificant with experimental data, hence polynomial regression of up to 9^th^ order was performed to achieve greater accuracy. It’s observed from the regression model that experimental and regression curves represent better fit with the achieved accuracy in the range of 95–99.99%. Fig 19 represents the exact fit curves of both experimental and regression values. Percentile Residual plot for all the samples varied with filler percentage of 0,1,3,5, & 7% for room temperature condition were represented from Fig 20. Percentile residual plots demonstrate the extent up to which how exactly these residuals fit with normal distribution. Presented plots depicts the correlation of actual residuals over expected normal distribution. Its observed from the plots that linearity trend is observed for all the tested samples shown in Fig 20 and it’s also noticed Fig 20a and d represent bit non-linear trend as actual residuals were slightly deviated from normal distribution which represents light tailed distribution as compared to other variations. Finally polynomial regression model of up to 9^th^ order was performed to achieve higher accuracy for all the room temperature aged samples. The present model helps to assess and validate the experimental data which is reference model to assess for other two sets of continuous heating and continuous cooling cycle temperatures.

## 4. Conclusion

The present work has made an attempt to validate filler influence on low velocity drop weight impact strength of GFRP composites along with temperature effect been investigated to know the impact characteristics through keeping the constant impact energy. Addition of filler with varied proportion affects greatly on drop weight impact behaviour of GFRP’s along with differed temperature values, It is concluded from the results that effect of filler contributes more towards improving energy absorption capability of samples start from 1 to 3% but beyond 3% the energy absorption range follows downtrend for further loading of filler indicating that more filler content leads to agglomeration in the matrix. It is concluded that, 3% wollastonite filled offers excellent resistance to impact load for all the temperature conditions of +50^0^c, −5^0^c including samples tested under room temperature conditions. Results reported nearly 18%,50%,33% increase in its value for 3% filler sample when compare to neat GFRP composites for the different temperature conditions of ambient, heating and cooling cycle respectively. The optimal filler loading percentage is concluded to be 3% which is same for all the temperature conditions. To conclude the temperature influence on Load taking capability for GFRP composite samples, Continuous heating cycle seems to be profound influencing on load taking ability of the composite where 3% filler loaded sample not only reports higher resistance to LVI loading but also reports excellent energy absorption for all the compositions subjected to different temperature ageing conditions, which reports the energy absorption range of 54–63% of which is about 31–54% higher value than that of neat composites for three temperature conditions. Additionally polynomial regression of order 9 was performed to understand the experimental correlation which is in good agreement. Fractography study reveals the matrix damage, fibre breakage, de-bonding of laminates witness the severity of damage mode observed when tested with different temperature conditions depicts. The conducted research explores the suitability of GFRP composites to be used for midterm exposure to low temperature to moderately high temperature with structural response against impact loading conditions. Developed, LVI tested composites may recommended to withstand bird strike impact, tool drops as well as any other maintenance-related impacts particularly under moderately high and low temperature conditions ensuring impact resistance which is critical in aerospace safety and performance under diverse environments.

## Supporting information

S1 Data **Fig 6.** Variation of Load over Displacement for different filler composition (Room temperature). **Fig 7.** Plot of Load over contact time for different filler composition (Room temperature). **Fig 9.** Variation of Load over Displacement for different filler composition (Heating cycle). **Fig 10.** Plot of Load over contact time for different filler composition (Heating cycle). **Fig 12.** Variation of Load over Displacement for different filler composition (Cooling cycle). **Fig 13.** Plot of Load over contact time for different filler composition (Cooling cycle). **Fig 16.** Influence of filler for 3 different temperatures. **Fig 17.** Influence of filler on different temperatures. **Fig 18.** Effect of filler and Temperature on Percentage of Energy Absorption. **Fig 19.** Correlation plots of experimental and regression values. **Fig 20.** Regression characteristics of room temperature tested samples.
(XLSX)
